# Autoimmune Hepatitis: Treatment Options and Management Review

**DOI:** 10.7759/cureus.15682

**Published:** 2021-06-16

**Authors:** Eyad Gadour

**Affiliations:** 1 Gastroenterology and Hepatology, University Hospitals of Morecambe Bay NHS Foundation Trust, Lancaster, GBR

**Keywords:** autoimmune hepatitis, liver cirrhosis, autoantibodies, azathioprine, hypergammaglobulinemia

## Abstract

Autoimmune hepatitis (AIH) is an inflammatory pathology of the liver which leads to liver cirrhosis and death if left untreated and affects a large population across the world with no ethnic discrimination. AIH can be asymptomatic or with non-typical clinical presentation. The diagnosis and categorization of AIH are based on the presence of autoantibodies, specific biochemical indices, and histopathological features. The categorization of AIH further supports therapeutic management decisions. Associated comorbidities are another worrisome in treatment decisions and better outcomes. Liver transplantation is the ultimate choice in case of zero or minimal therapeutic response or severe liver damage. Liver transplantation also has its associated risks and rejection concerns.

The international guidelines are designed to provide a complete management outline of AIH for better patient management. There is a disparity seen in these guidelines, especially in terms of dose recommendation. This review designed to lay out an overview of the new guidelines on the diagnosis and management of AIH.

## Introduction and background

Autoimmune hepatitis (AIH) is a chronic hepatic disorder with unresolved inflammation of liver cells because of an unidentified cause [1,2]. This continuous hepatic inflammation, if unmanaged, leads to liver cirrhosis and ultimately a liver failure, and responsible for the patient’s death [2]. The postulated theory of its pathogenesis proposed that environmental factors collapse human immunity and its tolerance activity, and collaboration of genomic susceptibility create a T cell-mediated immune reaction upon hepatic antigens, to create an uninterrupted fibrotic and necroinflammatory activity in the liver [3,4]. The substantial advancement of its management was reported between the 1970s and 1980s from England and the USA, based on clinical control trials [2]. AIH usually present with nonspecific clinical symptoms such as nausea, fatigue, abdominal pain, jaundice, and joint pain. The clinical presentation varied from nonspecific symptoms to asymptomatic presentation and acute severe liver disease [5-8]. The laboratory diagnosis may be helpful in association with clinical presentation such as the histopathological cellular pattern of liver cells, elevated serum globulin levels, presence of particular autoantibodies [1].

No vigorous epidemiological numbers are available of AIH in the United States. AIH is seen in all racial groups, however, seen more in women than men in a 3.6:1 ratio [1,9,10]. Some countries like Norway and Sweden reported their mean yearly incidence of 1 to 2 and 11 to 17 per 100,000 individuals, respectively [11-13]; European Association for the Study of the Liver (EASL) reported its prevalence in 15 to 25 cases per 100,000 individuals in Europe [14]. The previous scientific literature reported that about 40% of patients with unmanaged severe liver disease expire within six months of disease identification [13] and the living active patients of AIH developed further progression of severe liver-associated disorders such as cirrhosis and hemorrhage and in some cases esophagus-related disorders like esophageal varices [1]. An acute clinical symptomatic presentation of AIH is indicated by liver encephalopathy within eight weeks [1].

The management of AIH is still a crucial concern and after 50 years of efforts and trials, AHI is still one of the significant challenges of diagnosis and treatment. However, it was the first hepatic pathology for which treatment intervention of corticosteroid was strongly exhibited in clinical control trials. There are two principal reasons for this evident conflict: one, AIH is a quite rare disorder. Second, it is a heterogeneous disorder [14].

## Review

Sub-categorization of AIH

Autoantibody profile testing is an important tool for diagnostics, categorization, management, and therapeutic progress evaluation of AIH [1,2,14]. Sub-classification of AIH is mainly based on autoantibody profiles [14].

AIH-1

AIH-1 is the most common among all reported AIH cases and responsible for about 90% of AIH. The diagnostic tests which are useful for categorization and evaluation are antinuclear antibodies (ANA), smooth muscle antibodies (SMA), soluble liver antigen/liver pancreas antibodies (anti-SLA/LP), human leukocyte antigens (HLA), HLA DR3, DR4, and DR13, and histopathological cellular findings. AIH-1 usually well responds and well tolerated to AIH therapeutic agents with variable long-drug therapeutic requirements and relapse rates [14].

AIH-2

The diagnostic tests useful for categorization and evaluation are HLA DR3 anti-LKM1, liver/kidney microsomal antibody type 3 (anti-LKM-3), anti-LC1, antibodies, and liver/kidney microsomal antibody type 1 (anti-LKM-1). AIH-2 is usually seen in young children, adolescents, and teenage groups. Histopathological evaluations reported the degree of severity and advanced disease presentation. Treatment failure, relapse, and therapeutic withdrawals are frequently seen in this group and required long-term management therapy [14].

AIH-3

The criterion to distinguish AIH-3 from AIH-1 is positive results of SLA/LP and soluble liver antigen/liver pancreas antibodies. Another distinguishing characteristic is Ro52 (interferon-inducible protein) testing that is frequently seen positive in AIH-3 cases [14].

Hepatic disorders and association of AIH

AIH is linked with numerous different liver diseases, specifically cholestatic liver diseases and primary sclerosing cholangitis (PSC), drug-induced liver injury (DILI), viral hepatitis, and alcoholic or non-alcoholic steatohepatitis. Every condition has its own identification and management challenges [14]. The management of AIH is a specialized and crucial process and different specialized units with field competent experts have been designed and patients in specialized units have better treatment outcomes, in terms of survival rate and better quality of living [14].

AIH and management strategies

Different management guidelines are available to deal with this rare and complex disorder.

EASL Guidelines

Any patient with the acute or chronic liver disorder should be evaluated for AIH, especially with hypergammaglobulinemia, presence of autoantibodies, and specific cellular histopathological findings. A reliable, quick, and true diagnosis is the key to prevent high mortality and better management. Delayed and incorrect diagnosis leads to delayed management, especially for patients present with cirrhosis at initial examination. Another distinguishing feature of AIH in cirrhosis absence is elevated serum immunoglobulin G (IgG) levels without IgA and IgM levels elevation. This group of patients will be further monitored for fall of serum IgG levels for better treatment outcomes. AIH is usually linked with a wide range of different autoimmune disorders. All AIH children should go through MR cholangiography to eliminate the risk of autoimmune sclerosing cholangitis. Liver ultrasound for hepatocellular carcinoma (HCC) screening is recommended for all AIH cirrhosis patients in six months' duration. The immunosuppressant patient should undergo UV-protection counseling and dermatological monitoring to monitor the development of non-melanoma skin cancer [14].

AIH treatment should be focused to settle all biochemical and histopathological markers to avoid disease development in the future. The initial therapy for AIH was given by Prednisolon e0.5 and 1 mg/kg/day and added azathioprine after two weeks of Prednisolon. The treatment should be adjusted according to the advancement of the disease and only remission patients required close follow-up of three to six months; treatment may not be required in this case. Early treatment by intravenous corticosteroids (≥1 mg/ kg) in acute severe AIH patients is a recommended choice, in case of no improvement within seven days of treatment patient should be referred for an emergency liver transplant. An increase in the dose of prednisolone and azathioprine or alternative therapeutics should be used in case of the sub-optimal outcome. In case of low bilirubin levels 6 mg/dl (100 µmol/L) or two weeks after steroid treatment, azathioprine of 50 mg/day can be started; dose adjustment depends upon the patient’s response and toxicity effect. Azathioprine can be used up to 1-2 mg/kg. The core treatment concern of AIH should be response-dependent and followed on an individualized basis.

Immunosuppressive treatment is another approach and needs long-term management of approximately two to three years until normal levels of IgG and transaminases are achieved. Treatment should not be stopped if there is no remission seen by biochemical indices, and histopathology evaluation should be carefully analyzed in case of biochemical remission before treatment withdrawal decision. If pathological findings are seen on histology, therapy should be continued. A relapse is usually seen within 12 months of withdrawal but may occur even many years later. Lifelong surveillance is recommended to avoid any severe relapse outcome. Management of relapse requires the same induction regimen of steroids. Earlier relapse identification is managed by a low dose of immunosuppressants to persuade complete remission. Therapeutic intolerance of azathioprine is also seen in mild cases; prednisolone monotherapy is helpful in these cases. All other cases should be treated with azathioprine (mycophenolate mofetil). Childhood AIH is managed by a high dose of steroid, and the other management principle is the same as adult.

Vitamin D supplementation and increased calcium supplement should be suggested to all steroid-administered patients. In the absence of liver cirrhosis, budesonide and azathioprine combination are used as induction management and also in cases with co-morbidities association. The additional safety management is vaccine administration of hepatitis A, B, and influenza, to all AIH-diagnosed patients.

BSG Guidelines

British Society of Gastroenterology (BSG) guidelines classify AIH into two categories based on autoantibody testing: type 1 AIH and type 2 AIH. The characteristic autoantibodies of type 1 are ANA (negative for 25% of patients), ASMA anti-actin antibody, anti-SLA/LP antibodies, with rare treatment failure response [2]. Anti-LKM-1 and anti-LC-1 antibodies are the autoantibodies of type 2 AIH. Type 2 is usually seen in childhood and young adulthood with a 10:1 female/male ratio. It is a generally severe and advanced disease with high inflammatory cirrhosis. Treatment failure and relapse are common and long-term management is required in all cases.

Liver histology, laboratory features, serum immunoglobulins, serum autoantibodies, viral markers, and alcohol consumption are the diagnostic characteristics adapted from the International Autoimmune Hepatitis Group (IAIHG) [15].

Prednisolone plus azathioprine is the initial management recommendation of AIH; the current evidence is incapable to allow other therapeutics as initial management. Prednisolone 30 mg/day is recommended initially, which gradually reduces 10 mg/day up to four weeks with 1 mg/kg/day of azathioprine. Higher doses of prednisolone may also be used in initial management for quick recovery and normal levels of transaminases, but should not be used in frail aged patients. A higher steroid dose combined with azathioprine 2 mg/kg/day is advisable in non-responding and minimal responding patients. In some cases, tacrolimus can be used but with expert clinical advice. Liver transplant centers should be involved in cases with liver failure, bridging liver necrosis, and cases of unimproved model for end-stage liver disease (MELD) score.

AIH cases of prednisolone intolerance are treated by budesonide. Young patients with moderate or severe AIH having liver cirrhosis and histopathological features (even mild) should be administered immunosuppressive management. Calcium and vitamin D supplementation is also advised by BSG in AIH management. Bone DEXA screening is recommended every one to two years. Cases of steroid management should be carefully monitored for osteoporosis and manage accordingly in case of any minor signs seen. AIH patients who would not get remission by prednisolone and azathioprine treatment continued to treat with the same drug with a higher dose of 2 mg/kg/day and repeat liver biopsy after 12-18 months. Liver transplantation is the last option in AIH management and about 10-20% of AIH patients pursue this option. Liver transplantation is advisable in AIH patients with decompensation presentation, severe AIH with no improvement of transaminases after therapeutic intervention, fulminant liver failure, the MELD score of >15, and patients with HCC, meeting the transplantation criteria. Calcium and vitamin D supplementation, hepatitis A and B vaccination in susceptible AIH patients also advised.

American Association for the Study of Liver Diseases

The diagnostic criterion is the same as defined by the BSG guidelines. Based on serological markers, two types of AIH (types 1 and 2) have been defined. Corticosteroid is the absolute therapeutic indication [1].

Immunosuppressive management should be given to adult patients with 10-fold elevation of transaminases (ALT or AST) levels or more than fivefold of the upper limit of normal (ULN) and at least a twofold increase of ULN of gamma-glutamyl transferase (GGT) and/or the presence of histopathological findings such as bridging necrosis. However, Immunosuppressive management is not recommended in cases of AIH associated with other comorbidities like uncontrolled hypertension, psychosis, brittle diabetes, vertebral compression, and/or prednisone intolerances. Inert cases of AIH would only need close monitoring of three to six months and the use of immunosuppressive management is not recommended.

Initial treatment is the same with EASL and BSG guidelines by using prednisone and azathioprine. Adult cases of treatment failure should initially be managed by a high dose of prednisone and azathioprine. In case of no improvement, mycophenolate mofetil or cyclosporine is used for management (Table 1).

**Table 1:** Overview of therapeutic management of autoimmune hepatitis ESG: European Association for the Study of the Liver, BSG: British Society of Gastroenterology, AASLD: American Association for the Study of Liver Diseases.

Therapeutic management of AIH		
	Monotherapy Prednisone (mg/daily)	Combined Azathioprine
EASL 2014 [14]	0.5-1 mg/kg	Up to 1-2 mg/kg/daily
BSG 2011 [2]	30 mg/day	1 mg/kg/daily
AASLD (USA) 2010 [1]	60 mg daily to gradually decrease up to 20 mg daily	Prednisone: 30 mg daily to gradually decrease up to 10 mg daily azathioprine: 50 mg daily
AASLD (Europe (mg/kg/daily) [1]	60 mg daily to gradually decrease up to 20 mg daily	Prednisone: 30 mg daily to gradually decrease up to 10 mg daily azathioprine: 1-2 mg/kg/daily

Therapeutic cautions

Prednisone

Clinicians must include continuous assessment of ophthalmological abnormalities, and osteoporosis symptoms in all prednisone-treated AIH patients. Periodic evaluation checkups and vitamins and energy supplements lower the associated risk of prednisone administration [16,17].

Azathioprine

Leukocytopenia (decreased white cell count) and thrombocytopenia (decreased platelet count) should be monitored with azathioprine administration in all AIH patients [16].

Pregnancy and AIH

AIH and pregnancy is a very rare presentation but yet found in very small numbers especially in the postpartum period [14]. Azathioprine and glucocorticoids are generally considered safe therapeutics during pregnancy [14,16,18].

Liver Transplantation

In severe AIH cases, steroid therapy is advised and might prevent the risk of liver transplantation [14]. There are two main indications of liver transplantation; one is severe acute AIH which results in liver failure, the second indication is more critical due to decompensated chronic hepatic disease or hepatocellular carcinoma due to longstanding AIH [2].

De Novo Graft Dysfunction

"De novo" or "alloimmune hepatitis" or "graft dysfunction" may occur a long time or many years after grafting. This condition must be categorized separately from acute and chronic rejection, drug intoxication, and viral infection [19-21].

AIH management with liver co-morbidities

Non-alcoholic Fatty Liver Disease

Non-alcoholic Fatty Liver Disease (NAFLD) is the liver pathology that increases the progression of liver diseases. AIH and NAFLD both have persistently increased levels of transferases and autoantibodies detection. Corticosteroids are used to exacerbate other associated metabolic conditions such as diabetes, obesity, and hypertension. The lowest possible effective dose of corticosteroids is recommended for this group [14,22,23]. 

Hepatitis B and C Infection

Hepatitis B and C infection should not be considered at the time of AIH diagnosis; AIH may develop with HBV or HCV infection or in response to interferon therapy. In case of negative testing of HBV and HCV or no vaccination history, vaccination must be administered. In the case of AIH and viral hepatitis, an interferon-free regimen should be used initially and followed with immunosuppressive drugs [14,24]. 

Human Immunodeficiency Virus infection

Immunosuppressive therapeutic management should be used in AIH-HIV infections, and severe cases should be managed on an individualized basis [2].

AIH and Effects of Quality Care and Quality Life

Patient care and quality of life are the key factors in improved and better care. Health education and management care should be properly guided to the patient and their caretaker [2].

## Conclusions

With accurate diagnosis and timely management of AIH, patients can lead their well-managed and quality expectancy of living. Expert clinical management with standard and updated guidelines is the key to better patient care and disease elimination. The therapeutic dose disparity is seen in different standard guidelines, which must be resolved by clinical control trials to establish a transparent management protocol globally.
